# The comparative mitogenomics and phylogenetics of the two grouse-grasshoppers (*Insecta*, *Orthoptera*, *Tetrigoidea*)

**DOI:** 10.1186/s40659-017-0132-9

**Published:** 2017-10-05

**Authors:** Yufang Sun, Dianfeng Liu, Bo Xiao, Guofang Jiang

**Affiliations:** 1 Key Laboratory for Ecology and Pollution Control of Coastal Wetlands, School of Environmental Science and Engineering, Yancheng Institute of Technology, No.9, Yingbin Road, Yancheng, 224051 Jiangsu China; 2 School of Biology and Basic Medical Sciences, Medical College, Soochow University, 199 Ren’ai Road, Suzhou, 215123 Jiangsu China; 3 College of Oceanology and Food Science, Quanzhou Normal University, Quanzhou, 362000 Fujian China; 4 Department of Bioengineering and Food Engineering, Puyang Vocational & Technical Institute, Puyang, 457000 Henan China

**Keywords:** Mitogenome, *Orthoptera*, *Tetrigoidea*, Phylogenetic

## Abstract

**Objective:**

This study aimed to reveal the mitochondrial genomes (mtgenomes) of *Tetrix japonica* and *Alulatettix yunnanensis*, and the phylogenetics of *Orthoptera* species.

**Methods:**

The mtgenomes of *A. yunnanensis* and *T. japonica* were firstly sequenced and assembled through partial sequences amplification, and then the genome organization and gene arrangement were analyzed. Based on nucleotide/amino acid sequences of 13 protein-coding genes and whole mtgenomes, phylogenetic trees were established on 37 *Orthoptera* species and 5 outgroups, respectively.

**Results:**

Except for a regulation region (A+T rich region), a total of 37 genes were found in mtgenomes of *T. japonica* and *A. yunnanensis*, including 13 protein-coding genes, 2 ribosomal RNA genes, and 22 transfer RNA genes, which exhibited similar characters with other *Orthoptera* species. Phylogenetic tree based on 13 concatenated protein-coding nucleotide sequences were considered to be more suitable for phylogenetic reconstruction of *Orthoptera* species than amino acid sequences and mtgenomes. The phylogenetic relationships of *Caelifera* species were *Acridoidea* and *Pamphagoidea* > *Pyrgomorphoidea* > *Pneumoroidea* > *Eumastacoidea* > *Tetrigoidea* > *Tridactyloidea*. Besides, a sister-group relationship between *Tettigonioidea* and *Rhaphidophoroidea* was revealed in *Ensifera*.

**Conclusion:**

Concatenated protein-coding nucleotide sequences of 13 genes were suitable for reconstruction of phylogenetic relationship in orthopteroid species. *Tridactyloidea* was a sister group of *Tetrigoidea* in *Caelifera*, and *Rhaphidophoroidea* was a sister group of *Tettigonioidea* in Ensifera.

**Electronic supplementary material:**

The online version of this article (doi:10.1186/s40659-017-0132-9) contains supplementary material, which is available to authorized users.

## Introduction

Mitochondrial genome (mtgenome) is a kind of small circular molecule in most of metazoans, which evolves semi-independently from nuclear genomes and plays an important role in the process of metabolism, programmed cell death, illness, and aging. Generally, the closed circular mtDNA was 14–39 kb in length, which consists of a major non-coding region (regulation region, A + T rich region) and a canonical set of 37 genes, including 13 protein-coding genes, 2 ribosomal RNAs (rRNA) and 22 transfer RNAs (tRNA). The distribution of these genes is always compact with infrequent introns and intergenic space [1, 2]. As low frequency of intermolecular genetic recombination and relatively rapid evolutionary rate, mtgenome has been extensively used for researching on population structures, phylogeography and phylogenetic relationships at various taxonomic levels [3, 4].

Recently, mtgenome has been widely used in phylogenetic analyses. It has been reported, mtgenomes could provide rich information’s in phylogenetics [5]. Phylogenetic analyses based on complete mtgenome sequences could improve the statistical confidence of inferred phylogenetic trees with better resolution than analyses only based on partial mtgenes [6]. The evolution of mtgenomes, instead of mtgenes, was a new instrument for studying biological speciation and lineage divergence [7]. In addition, mtgenome may partly represent the whole genome, and be used as a phylogenetic marker in investigation of structural genomic features easily and systematically [8]. All these features of mtgenome greatly promoted the researches on evolutionary trends and relationships of phylogenetically distant organisms [9].

With the growing interest in mtgenomes, a rapid increase of published complete mtgenome sequences was revealed [10]. Despite insects were the most species-rich class animals, the sequenced mtgenomes are majorly vertebrates. Until now, more than 8634 complete metazoan mtgenomes have been sequenced, and only 337 are from insects and 39 are from *Orthoptera* (http://www.ncbi.nlm.nih.gov). Besides, two mtgenomes of *Tetrigoidea* were announced by our previous studies [10]. *Orthoptera* is a kind of primitive hemimetabolous insects, contains approximately 20,000 described species in two suborders of equal size (*Caelifera* and *Ensifera*) [11]. A preliminary phylogenetic analyses of *Orthoptera* based on the mtgenome data have been performed, while the superfamily *Tetrigoidea* was not involved. *Tetrigoidea* is a moderately diverse group of basal *Caelifera* comprising approximately 1400 species in 8 families and 270 genera [12]. As a monophyletic group supported by molecular data, *Tetrigoidea* was regarded as one of the oldest groups in *Caelifera*, which closely related to *Tridactyloidea* [13, 14]. Researches on the mtgenome sequences of *Tetrigoidea* may contribute to the revelation of phylogenetic relationships in *Orthoptera*. In this study, the mtgenomes of two *Tetrigoidea* species, *A. yunnanensis* and *T. japonica* were firstly revealed, and the genome organization and gene arrangement were then analyzed. Meanwhile, phylogenetic trees were established to evaluate the phylogenetics of *Orthoptera* species. Our findings may enrich our knowledge on mtgenomes of *Tetrigoidea*, and provide an efficient strategy for biodiversity exploring on *Orthoptera* species.

## Materials and methods

### Samples and DNA extraction

Specimens of *A. yunnanensis* and *T. japonica* were collected from a public land (not a protected area or a national park) in Nanjing, Jiangsu, China. Total genomic DNA was extracted from the femoral muscle of fresh specimens by the standard proteinase K and phenol/chloroform extraction method. Simply, the tissues were firstly disintegrated with 20 mg/ml proteinase K (Genebase Gene-Tech Co., Ltd) at 37 °C for 2–3 h. Then, the samples were incubated with extraction solution, and V/2 of phenol and V/2 of chloroform was added. After centrifugation, the supernatant was obtained, and 1/10 volume of 3 M NaOAc and 2 volumes of 100% ethanol were used to precipitate the DNA. Finally, the precipitate (DNA) was dissolved in Tris–EDTA buffer solution, and quantified with spectrafluorometer. The isolated DNA samples were stored at −20 °C and used as a template for subsequence PCR reactions.

### Primer design and PCR amplification

Some partial sequences were firstly amplified and sequenced using general primers based on Simon et al. [15]. Then, new primers were designed based on determined sequences, and each amplified segments could overlap the adjacent segments (Primers were shown in Table 1). The fragments of mtgenomes were amplified by PCR using Takara LA Taq™ (Takara Bio, Otsu, Shiga, Japan). The PCR program included an initial denaturation at 94 °C for 3 min, followed by 10 cycles of denaturation at 94 °C for 30 s, annealing at 52–59 °C to 0.3 °C/cycle (depending on primer combinations) for 30 s, elongation at 68 °C for 60–180 s (depending on putative length of the fragments); then followed by another PCR program included 20 cycle of 30 s denaturation at 94 °C, 30 s annealing at 49–56 °C, 60–180 s elongation at 68 °C and a final extension at 68 °C for 8 min. The PCR products were identified by electrophoresis on 1% agarose gel.

**Table 1:** Sequencing primers used in the analysis of mitochondrial genome of *Alulatettix yunnanensis* and *Tetrix japonica*

Upstream primers sequences (5′–3′)	Downstream primers sequences (5′–3′)	Anneal temperature (°C)
190-J: AAGCTAMTGGGTTCATRCCC	1650-F: AAYCAATTTCCGAATCCACC	53
1600-J: GTTGTTGTAACAGCACATGC	2750-F: CCTCCTATAATAGCAAATACTGCTCC	54
2650-J: TTACCTGTTYTWGCWGGAGC	3660-F: CCACAAATTTCAGAGCATTGACC	55
3600-J: CAATGATACTGATCATATGAATATTC	4900-F: ATCYCGTCATCATTGAATTAT	53
4800-J: TAGTAGACTATAGTCCATGACC	6150-F: CCATTCTTTCAGGTCGAAACTG	55
5800-J: GAGCAWCTTAGGGTTATAGTT	7600-F: TAAGWAATCGKRTWGGTGATGT	52
7500-J: CAGGAGTAGGAGCAGCTATAGC	8650-F: CTTGTAATATATCGGCTCCTCC	56
8500-J: GTGTAATAAGAATAACTAATTAAGCC	9000-F: TGTTGCAGCTTCATTACCATTATTGT	49
8900-J: GGGGCCTCAACATGAGCYTT	10600-F: TTTCATCATATTGAAATRTTTRTTGG	51
10300-J: CAACAATAATGAAACAAYRAATATAG	11600-F: AAATAYCATTCTGGTTGAATGTG	51
11450-J: CCCATATATTATAGGAGAYCC	12300-F: TATGAGTTCGGGGTACTTTACC	53
12050-J: AAAAACCCCCTTCAAGCCAAAT	13350-F: GACYGTRCAAAGGTAGCATAATC	54
13150-J: TTCTCGTTAAACCTTTCATTCCAGT	14300-F: TATTTCAGGTCAAGGTGCAGCTTAT	54
14100-J: CTACTWTGTTACGACTTATCTC	14450-F: ARACTAGGATTAGATACCCT	51
14330-J: TAACATCATTCATGAAACAGGTTCCTCT	250-F: ATTTCTAGTCCTATTCACACACCTAATC	54

### Sequencing and sequence assembly

The PCR products with single band were purified using a V-gen PCR clean-up purification kit. If more than one band was present, the appropriately sized PCR product was cut off from the gel and purified using a biospin gel extraction kit. All fragments were sequenced in both directions, and some PCR products were sequenced by primer walking strategy. The identified sequences were assembled by seqman (DNASTAR 2001), BioEdit and Chromas 2.22, and then the complete mtgenome sequences of *T. japonica* and *A. yunnanensis* were manually checked. The coverage of each mtgenome was above two times.

### Sequence analysis

Gene encoding proteins, rRNA and tRNA were identified according to their amino acid translation or secondary structure features, respectively. Individual gene sequences were compared with the available homologous sequences of *Orthoptera* species in GenBank. A total of 22 *tRNA* genes were identified using software *tRNA* Scan-SE 1.21 (http://lowelab.ucsc.edu/tRNAscan-SE) and their cloverleaf secondary structures and anticodon sequences were identified using DNASIS (Ver.2.5, Hitachi Software Engineering).

### The reconstruction of phylogenetic trees

In order to evaluate the phylogenetic relationships in *Orthoptera*, phylogenetic trees were established based on nucleotide/amino acid sequences of 13 protein-coding genes and whole mtgenome sequences of 37 *Orthoptera* species whose complete mtgenome sequences were available in GenBank by using two *Blattaria* species (*Periplaneta fuliginosa* and *Eupolyphaga sinensis*), two Isoptera specie (*Reticulitermes flavipes* and *Coptotermes formosanus*) and one Mantodea specie (*Tamolanica tamolana*) as outgroup [6]. Mtgenome sequences were downloaded from GenBank (Table 2).

**Table 2:** A total of 37 *Orthoptera* species were used in reconstruction of phylogenetic trees. Two *Blattaria* species, two *Isoptera* specie and one *Mantodea* specie were considered as outgroup

Taxa	Species	Accession
*Caelifera*/*Tetrigoide*a	*Tetrix japonica*	
	*Alulatettixyunnanensis*	JQ272702
*Caelifera*/*Acridoidea*	*Acridacinerea*	GU344100
	*Acridawillemsei*	EU938372
	*Arcypteracoreana*	GU324311
	*Chorthippuschinensis*	EU029161
	*Euchorthippusfusigeniculatus*	HM583652
	*Gastrimargusmarmoratus*	EU513373
	*Gomphocerussibiricustibetanus*	HM131804
	*Gomphoceruslicenti*	GQ180102
	*Locustamigratoriatibetensis*	HM219224
	*Locustamigratoria*	X80245
	*Oedaleusdecorusasiaticus*	EU513374
	*Ognevialongipennis*	EU914848
	*Oxyachinensis*	EF437157
	*Phlaeobaalbonema*	EU370925
	*Prumnaarctica*	GU294758
	*Schistocercagregariagregaria*	GQ491031
	*Trauliaszetschuanensis*	EU914849
	*Xyleusmodestus*	GU945503
*Caelifera*/*Eumastacoidea*	*Pielomastaxzhengi*	JF411955
*Caelifera*/*Pamphagoidea*	*Thrinchusschrenkii*	GU181288
*Caelifera*/*Pneumoroidea*	*Physemacrisvariolosa*	GU945504
*Caelifera*/*Pyrgomorphoidea*	*Atractomorphasinensis*	EU263919
	*Mekongiellaxizangensis*	HM583654
	*Mekongianaxiangchengensis*	HM583653
*Caelifera*/*Tridactyloidea*	*Ellipesminuta*	GU945502
*Ensifera*/*Tettigonioidea*	*Anabrus simplex*	EF373911
	*Deracanthaonos*	EU137664
	*Elimaeacheni*	GU323362
	*Gampsocleisgratiosa*	EU527333
	*Ruspoliadubia*	EF583824
*Ensifera*/*Grylloidea*	*Gryllotalpaorientalis*	AY660929
	*Gryllotalpapluvialis*	EU938371
	*Myrmecophilusmanni*	EU938370
	*Teleogryllusemma*	EU557269
*Ensifera*/*Rhaphidophoroidea*	*Troglophilusneglectus*	EU938374
*Blattaria*	*Periplanetafuliginosa*	AB126004
	*Eupolyphagasinensis*	FJ830540
*Isoptera*	*Reticulitermesflavipes*	EF206314
	*Coptotermesformosanus*	AB626145
*Mantodea*	*Tamolanicatamolana*	DQ241797

### Alignments and bayesian analyses

The nucleotide and amino acid sequences were aligned by ClusterW in MEGA 4.0 with manual refinements [16]. One alignment was based on the complete mtDNA sequences, except for the highly variable ETAS (extended termination associated sequence) domain within regulation region, creating a sequence of 15,612 nt positions. The second alignment was based on the complete set of codons (except stop codons) creating a concatenated sequence of 10,989 nt positions (3663 amino acid positions) corresponding to the 13 protein-coding genes.

Bayesian analyses were performed by MRBAYES 3.1.2, with gaps treated as missing data [10]. The best fitting substitution model judged by Akaike information criterion (AIC) was determined by MrMODELTEST 2.3 [17]. For each BI analysis, two independent sets of monte carlo markov chains (MCMC) were run, each with one cold and three heated chains for 1 × 10^6^ generations, and every 1000 generations were sampled. The burn-in parameter was estimated by plotting-ln*L* against the generation number using TRACER v1.4.1, and the retained trees were used to estimate the consensus tree and Bayesian posterior probabilities [18].

## Results

### Genome organization and gene arrangement

By sequencing and sequence assembly, a total of 37 genes were found in mtgenomes of *T. japonica* and *A. yunnanensis*, including 13 protein-coding genes (*nad2*, *COI, COII, atp8, atp6, COIII, nad3, nad5, nad4, nad4L, nad6, cob and nad1*), 2 rRNA (*12S rRNA* and *16S rRNA*), and 22 tRNA. Meanwhile, a regulation region (A+T rich region) was also found in the mtgenomes (Table 3).

**Table 3:** Annotation of the mitochondrial genomes in *Tetrix japonica* (Tj) and *Alulatettix yunnanensis* (Ay)

Feature	Strand	Position		Initiation codon/Stop codon		Anticodon
		Tj	Ay	Tj	Ay	
*trnI*	J	1–64	1–65			GAT
*trnQ*	N	65–133	66–134			TTG
*trnM*	J	134–201	135–202			CAT
*nad2*	J	201–1202	203–1204	ATG/TAA	ATG/TAA	
*trnW*	J	1201–1266	1203–1268			TCA
*trnC*	N	1259–1324	1261–1326			GCA
*trnY*	N	1325–1388	1327–1390			GTA
*COI*	J	1386–2924	1388–2926	ATC/TAA	ATC/TAA	
*trnL*(UUR)	J	2920–2983	2922–2985			TAA
*COII*	J	2984–3667	2986–3669	ATG/TAA	ATG/TAA	
*trnD*	J	3666–3729	3668–3729			CTT
*trnK*	J	3730–3797	3730–3797			GTC
*atp8*	J	3802–3957	3802–3957	ATG/TAA	ATG/TAA	
*atp6*	J	3951–4622	3951–4622	ATG/TAA	ATG/TAA	
*COIII*	J	4625–5428	4625–5428	ATA/TAA	ATA/TAA	
*trnG*	J	5412–5474	5412–5474			TCC
*nad3*	J	5472–5828	5472–5828	ATA/TAG	ATA/TAG	
*trnA*	J	5827–5891	5827–5891			TGC
*trnR*	J	5891–5953	5891–5953			TCG
*trnN*	J	5950–6013	5950–6013			GTT
*trnS*	J	6013–6081	6013–6081			GCT
*trnE*	J	6081–6144	6081–6144			TTC
*trnF*	N	6143–6205	6143–6205			GAA
*nad5*	N	6207–7922	6206–7925	ATG/TAA	ATG/T–	
*trnH*	N	7926–7989	7929–7992			GTG
*nad4*	N	7989–9314	7992–9317	ATG/TAG	ATG/TAG	
*nad4L*	N	9308–9598	9311–9601	ATT/TAA	ATT/TAA	
*trnT*	J	9601–9666	9604–9668			TGT
*trnP*	N	9667–9730	9669–9732			TGG
*nad6*	J	9732–10,226	9734–10,228	ATG/TAA	ATG/TAA	
*cob*	J	10,226–11,362	10,228–11,364	ATG/TAG	ATG/TAG	
*trnS*(UCN)	J	11,361–11,428	11,363–11,430			TGA
*nad1*	N	11,441–12,385	11,443–12,387	ATA/TAA	ATA/TAA	
*trnL*	N	12,380–12,442	12,382–12,444			TAG
*16S*	N	12,443–13,739	12,445–13,784			
*trnV*	N	13,741–13,811	13,786–13,857			TAC
*12S*	N	13,812–14,597	13,858–14,644			
*A*+*T*−*rich*		14,598–15,128	14,645–15,104			

*J* represents sense strand, *N* represents antisense strand

The arrangement of mtgenome was very compact in these two species, which exhibited many gene overlaps. In *T. japonica*, 21 gene overlaps in 1–17 bp with a total of 77 bp in length were found. Similarly, 19 gene overlaps in 1–17 bp with a total of 75 bp in length were found in *A. yunnanensis*. In addition, 8 non-coding regions in 1–12 bp with a total of 26 bp in length, and 7 non-coding regions in 1–12 bp with a total of 25 bp in length were revealed in A+T-rich regions of *T. japonica* and *A. yunnanensi*s, respectively. Besides, 22 *tRNA* genes were also found in mtgenomes of *T. japonica* and *A. yunnanensi*s, which exhibited a same relative genomic position in other *Orthoptera* insects. The predicated secondary structures of these 22 *tRNA* genes in *T. japonica* and *A. yunnanensis* were shown in Additional file 1: Figure S1 and Additional file 2: Figure S2.

The nucleotide composition of these two mitogenomes (*T. japonica* and *A. yunnanensis*) biased toward adenine and thymine (75.57% in *T. japonica* and 75.24% in *A. yunnanensis*). ATN was the preferred initiation codon of 13 protein-coding genes in *T. japonica* and *A. yunnanensis*, including 8 ATG, 3 ATA, 1 ATC and 1 ATT. TAA and TAG were considered to be the termination codons of these 13 protein-coding genes in *T. japonica* and *A. yunnanensis*, except one T of *nad5* gene in *A. yunnanensis* (Table 3). Besides, the A+T-rich regions of the two mtgenomes were also located between small rRNA and *tRNA* ^*Ile*^, which were 531 bp with 82.67% A+T and 460 bp with 80.87% A+T in *T. japonica* and *A. yunnanensis*, respectively. Short repeating sequences except Poly A and Poly T could not be found throughout the whole A+T-rich regions.

### Phylogenetic analyses

**Fig. 1:**
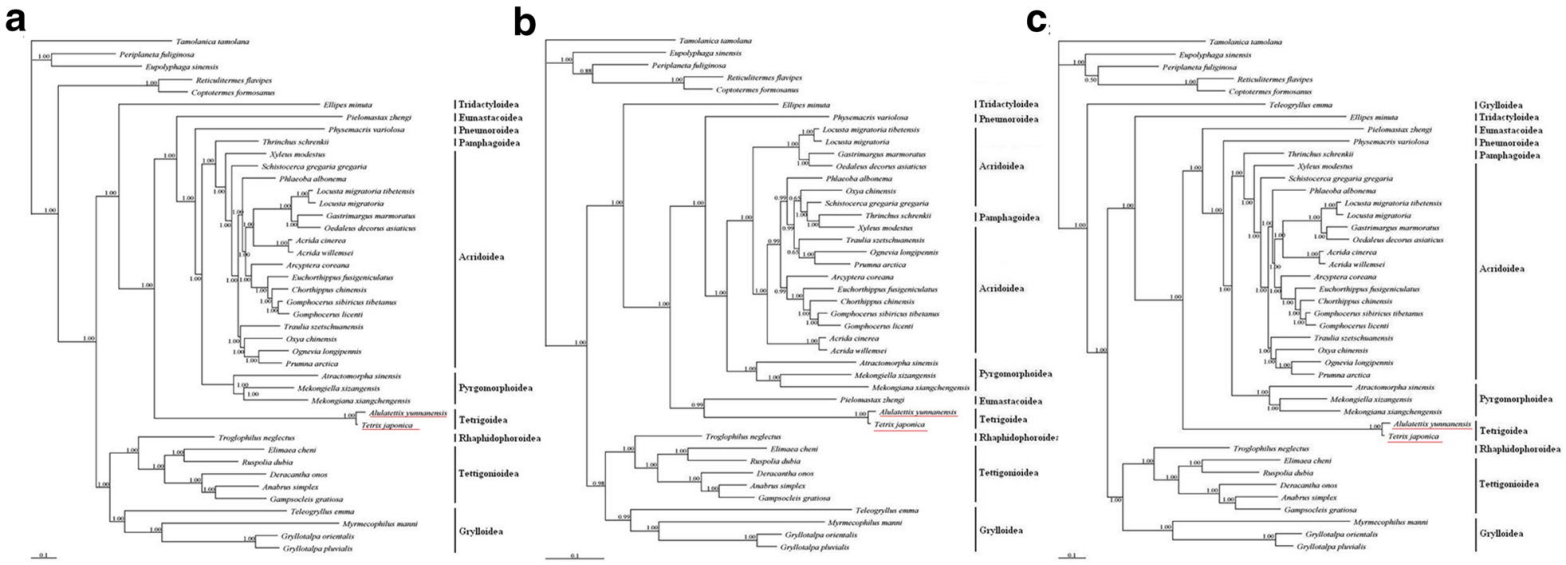
Phylogenetic tree established by concatenated protein-coding DNA sequences (N = 13) **a**, concatenated amino acids **b**, and whole mtgenome sequences **c** of 37 Orthopteran species and 5 outgroups (two *Blattaria* species, two *Isoptera* specie and one *Mantodea* specie). The red underline is the species position of *Alulatettix yunnanensis* and *Tetrix japonica*

Based on 13 concatenated protein-coding nucleotide sequences, the topology of established phylogenetic tree was similar with the reconstructed tree based on the whole mtgenome sequences. Differently, *Teleogryllus emma* of *Gryllidae* was revealed to be basal to all other Orthoptera species in phylogenetic tree of protein-coding nucleotide sequences, which was conflicted with the monophyletic *Gryllidae* in phylogenetic tree of mtgenome (Fig. 1a, c). In phylogenetic tree based on amino acid, *Thrinchus schrenkii* was found to belong to *Pamphagoidea* among various species of *Acridoidea*, which was also not consistent with the monophyletism of *Acridoidea* (Fig. 1b). According to the 37 *Orthoptera* species, 13 concatenated protein-coding DNA sequences were suspected to be accurate and effective for phylogenetic reconstruction of *Orthoptera* species.

As shown in Fig. 1a, two Orthopteran suborders, *Caelifera* and *Ensifera* were both recovered as monophyletic groups. In *Caelifera* branch, *Acridoidea*, *Pyrgomorphoidea* and *Tetrigoidea* were monophyletic groups. The phylogenetic relationships of these superfamilies were *Acridoidea* and *Pamphagoidea* > *Pyrgomorphoidea* > *Pneumoroidea* > *Eumastacoidea* > *Tetrigoidea* > *Tridactyloidea*. In *Ensifera*, a sister-group relationship between *Tettigonioidea* and *Rhaphidophoroidea* was revealed.

## Discussion

According to our previous studies, the mtgenomes of *T. japonica* (15,128 bp) and *A. yunnanensis* (15,104 bp) were circular molecules (GenBank accession numbers: JQ340002 and JQ272702) [19, 20]. In this study, a total of 37 typical genes and a regulation region were found in the mtgenomes of *T. japonica* and *A. yunnanensis*, which exhibited similar gene order and orientation with other Orthopteran insects. The conserved mtgenome structure in divergent insects identified their close genetic relationships [10]. In addition, the main nucleotide composition of these two mtgenomes was revealed to be adenine and thymine (75.57% of *T. japonica* and 75.24% of *A. yunnanensis*). Although the nucleotide composition was slightly lower than that found in some other *Orthoptera* insects (*Locusta migratoria* 75.3%, *Oxya chinensis* 75.9% and *Acrida willemsei* 76.2%), it was still corresponded well to the normal range of insect mtgenomes from 69.2% to 84.9% [10]. These data should be useful for developing mtgenome genetic markers for species identification of *Orthoptera* insects.

In mtgenomes of *T. japonica* and *A. yunnanensis*, 22 *tRNA* genes were identified in the same relative genomic positions as observed in other *Orthoptera* insects. The typical cloverleaf secondary structures and anticodons of these tRNAs were also similar to those found in other metazoan animals. As the only major non-coding region in insect mtgenome, the regulation region (A+T rich region) biased on A+T nucleotides were evolved under a strong directional mutation pressure [21]. It has been reported the A+T rich region was varied greatly in insects, from 70 bp in *Ruspolia dubia* to 4601 bp in *Drosophila melanogaster* [22, 23]. In this study, A+T rich regions in 531 bp length with 82.67% A+T and 460 bp length with 80.87% A+T located between small rRNA and *tRNA* ^*Ile*^ were revealed in *T. japonica* and *A. yunnanensis*, respectively. This region may limit its use for both inter- and intra-specific analyses in evolutionary studies.

In phylogenetic analyses, a similar topology of the established phylogenetic trees based on the whole mtgenome sequences and concatenated protein-coding nucleotide sequences were revealed. However, *Teleogryllus emma* of *Gryllidae* basal to all other *Orthoptera* species based on nucleotide sequences was conflict with the monophyletic *Gryllidae* based on mtgenome sequences. This phenomenon may be explained by that the mitochondrial non-protein-coding sequences of *Orthoptera* species, such as tRNA genes with nucleotide conservation were different from protein-coding sequences with relatively fast evolutionary rate, thereby disturbing phylogenetic reconstruction [24]. In addition, the phylogenetic tree based on amino acid showed that *Thrinchus schrenkii* of *Pamphagoidea* was nested within *Acridoidea*, which was conflicted with the monophyletism of *Acridoidea*. As amino acid sequences were usually conserved due to invisible synonymous substitutions in amino acid level, nucleotide sequences may be more reliable for phylogenetic reconstruction of closely related *Acridoidea* species [25]. These results of phylogenetic trees in 37 Orthopteran species indicated that the best way for phylogenetic reconstruction of *Orthoptera* was based on the concatenated protein-coding nucleotide sequences, but not the amino acid sequences and entire mtgenomes. As shown in phylogenetic trees based on concatenated protein-coding nucleotide sequences, two Orthopteran suborders, *Caelifera* and *Ensifera*, were both recovered as monophyletic groups, which were consisted with previous studies of morphological and molecular data [5]. The phylogenetic relationships of the superfamilies in *Caelifera* also supported previous results of Flook and Rowell [13]. Besides, a sister group relationship between *Tettigonioidea* and *Rhaphidophoroidea* was revealed in *Ensifera*, which was also consist with the results presented by Fenn et al. [5] and Zhou et al. [26]. The assumption that *Gryllidae* was basal to all other *Ensifera* received strong supports.

In conclusion, *T. japonica* and *A. yunnanensis*, together with other *Orthoptera* species, exhibited the same mitochondrial genome organization. The concatenated nucleotide sequences of 13 protein genes were suitable markers for reconstruction of phylogenetic relationship in *orthopteroid* species. The relationships of *Tridactyloidea* as sister group of *Tetrigoidea* in *Caelifera* and *Rhaphidophoroidea* as sister group of *Tettigonioidea* in *Ensifera* were identified. However, this study was still limited by insufficient species, and their phylogenetic relationships were not accurately identified. Further researches on mtgenome data and morphological characters were still needed to reveal the relationships of *Orthoptera* species.

## Additional files

**Additional file 1: Figure S1.** Predicated secondary structure of the 22 tRNA genes of *Tetrix japonica*.

**Additional file 2: Figure S2.** Predicated secondary structure of the 22 tRNA genes of *Alulatettix yunnanensis*.
