# Supplementary figures and images for: The comparative mitogenomics and phylogenetics of the two grouse-grasshoppers (Insecta, Orthoptera, Tetrigoidea)
Source: Biol Res. 2017 Oct 5;50:34. doi: 10.1186/s40659-017-0132-9 (PMC5629798; doi:10.1186/s40659-017-0132-9)

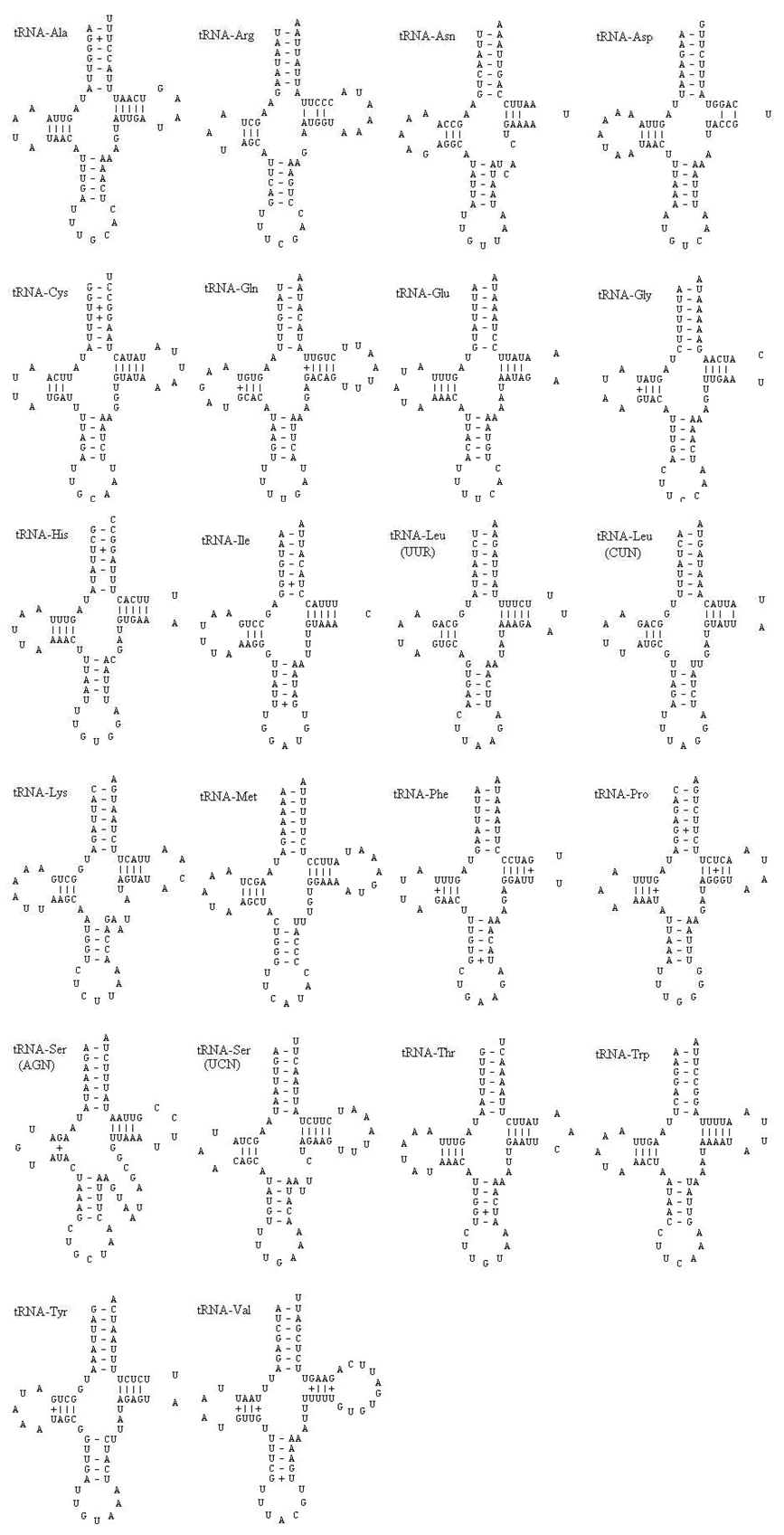

Supplement: Supplementary file 1 — Additional file 1: Figure S1. Predicated secondary structure of the 22 tRNA genes of Tetrix japonica. [file 40659_2017_132_MOESM1_ESM.tif]

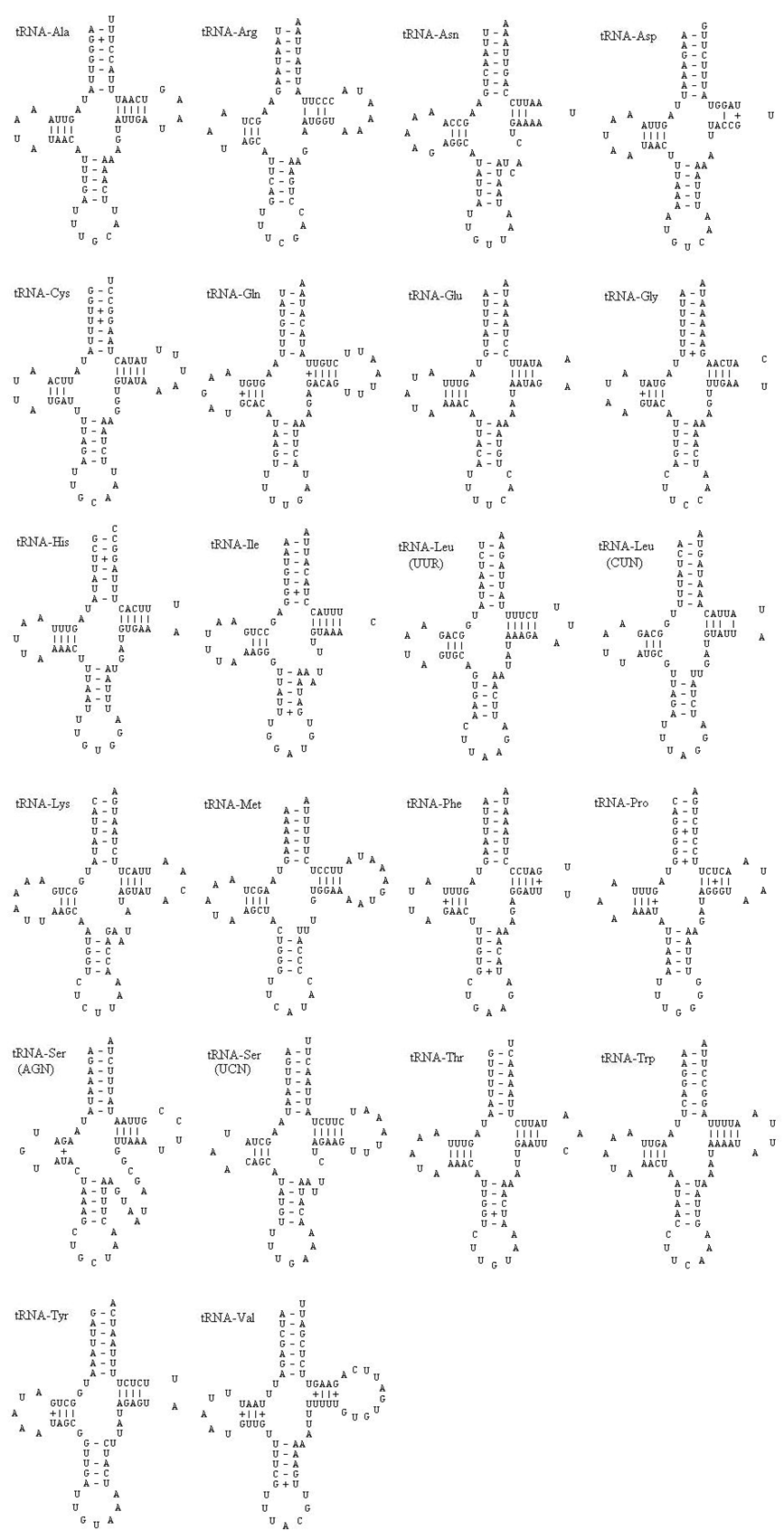

Supplement: Supplementary file 2 — Additional file 2: Figure S2. Predicated secondary structure of the 22 tRNA genes of Alulatettix yunnanensis. [file 40659_2017_132_MOESM2_ESM.tif]
